# Alterations with Movement Duration in the Kinematics of a Whole Body Pointing Movement

**DOI:** 10.1371/journal.pone.0052477

**Published:** 2013-01-14

**Authors:** Matthieu Casteran, Patrick Manckoundia, Thierry Pozzo, Elizabeth Thomas

**Affiliations:** 1 UFR-STAPS, INSERM U-1093, Cognition, Action and Sensorimotor Plasticity Université de Bourgogne, Campus Universitaire, Dijon, France; 2 Service de Médecine Interne Gériatrie, Hôpital de Champmaillot, Centre Hospitalier Universitaire, Dijon, France; 3 Italian Institute of Technology, Genoa, Italy; 4 Institut Universitaire de France, Paris, France; The University of Western Ontario, Canada

## Abstract

Our aim was to investigate how the organization of a whole body movement is altered when movement duration (MD) is varied. Subjects performed the same whole body pointing movement over long, normal and short MDs. The kinematic trajectories were then analyzed on a normalized time base. A principal components analysis (PCA) revealed that the degree of coordination between the elevation angles of the body did not change with MD. This lack of significant differences in the coordination was interesting given that small spatial and temporal differences were observed in the individual kinematic trajectories. They were revealed by studying the trajectories of the elevation angles, joint markers and center of mass. The elevation angle excursions displayed modifications primarily in their spatial characteristics. These alterations were more marked for the short rather than long duration movements. The temporal characteristics of the elevation angles as measured by the time to peak of angular velocity were not modified in the same fashion hence displaying a dissociation in the tuning of the spatial and temporal aspects of the elevation angles. Modifications in the temporal characteristics of the movement were also studied by examining the velocity profiles of the joint markers. Interestingly, unlike the disordered nature of this variable for the elevation angles, the time to peak velocity was neatly ordered as a function of MD for the joint markers – It arrived first for the short duration movements, followed by those of the normal and finally long duration movements. Despite the modifications observed in the kinematic trajectories, a PCA with the elevation angle excursions at different MDs revealed that two principal components were sufficient to account for nearly all the variance in the data. Our results suggest that although similar, the kinematic trajectories at different MDs are not achieved by a simple time scaling.

## Introduction

One of the fundamental features that can be adjusted in any movement is its duration. In this study we examine how the kinematics of a whole body movement is adjusted for different movement durations (MD). Several studies exist on the effects of movement duration on the kinetics and kinematics of arm movements [1], [2], [3], [4], [5], [6], [7], [8], [9], [10]. Theoretical work has been done to show the independance from MD of their velocity profiles and their movement path during reaching [2], [10]. Experimental work has confirmed this invariance [1], [3]. Further work has shown that the kinematic and muscular activation features reflect a strategy for arm movements that that is more in keeping with optimizing dynamic forces rather than minimizing antigravity torques [8], [6]. While the study of arm movements has provided us with much insight into the organization of movement, many of our daily activities associate focal displacements with simultaneous postural demands. Whole body pointing movements are therefore interesting to study. The trunk is a much heavier segment than the arm. Reaching over could bring into play a greater role for the gravitational component, changing the forces that are optimized and hence could reorganize the kinematics of the whole body pointing.

There are several more immediate reasons for carrying out the current study. Previous research has been done on the effects of MD on whole body pointing. This research however was restricted to examining the effects of decreasing movement duration (MD), i.e. at higher movement speeds [11], [12], [13], [14]. The previously cited studies showed that the adjustments of the whole body pointing movements for short MDs, was not achieved by a simple time scaling. In this study, we extend this research to look into the results of the opposite process, i.e. increasing MD. There are many reasons for doing this. Firstly slow movements are more subject to various types of modifying processes that have the potential for changing their trajectories. One of these is sensorial and especially proprioceptive feedback. An examination of the EMGs of slow arm movements therefore reveals trajectories that are not as smooth as those from normal or fast movements [4], [15]. This feedback is also probably the source of a greater variability that is frequently observed in slow movements [16]. Another potential source of modifications in slow movements is their relationship to gravity. Especially for moving downwards, slowing down must involve the use of force to counter the normal gravity dictated speed of the body. Nishikawa et al. [8] in studies of arm movements over long durations did not observe differences in the way the movement was organized. But would this observation extend to movements involving heavier segments of the body? An answer to this question would allow us to contribute to the picture on ‘speed sensitive strategies’ [4], [17]. It would also permit us to have a control for studies in which long MD could be a confounding factor. One important example of this is studies on ageing where it is always necessary to ask if the observed effects are due to anything more than the lower speeds with which elderly subjects perform most movements [18], [19], [20], [21].

The current investigation was carried out by examining the kinematics of a whole body pointing movement executed over long, normal and short durations. An examination of the individual kinematic trajectories as well as their degree of coordination with each other was carried out. The kinematic trajectories compared at the different MDs were the elevation angles, the joint marker positions and the center of mass (CoM) trajectory. A visual examination of the eight elevation angle trajectories revealed that the movements were carried out using a similar strategy in each case. This similarity was further quantified with the use of correlation coefficients once the dimensionality of the space had been reduced using the principal components analysis technique. This procedure revealed elevation angle excursions at different MDs that were similar albeit with small differences. The application of the PCA also allowed us to compare the degree of covariation among the elevation angles at each MD. It was not found to be significantly altered by MD. As the previously described analyses had revealed small differences in the kinematic trajectories executed over different durations, we carried out a detailed comparison of them. For the elevation angles we compared the amplitudes and the time to peak of their velocity profiles. At levels that involved more integrated effects, we examined the temporal characteristics of the joint marker velocity profiles and the spatial characteristics of the CoM trajectory. It should be noted that the comparisons of all trajectories were done using a normalized time base. Despite the differences observed in the kinematic trajectories, PCA analyses, this time using trajectories from different MDs revealed that two common waveforms could be combined to produce the movement trajectories at different MDs. This therefore provides a means for reducing the number of degrees of freedom for whole body pointing [22].

## Materials and Methods

### Participants

Eleven healthy adults, 3 women and 8 men (mean age: 26±6 years; mean height: 1.73 m±0.08 m; mean weight: 66±11 kg) took part in the experiments. None of the subjects had any previous neurological diseases and they had normal or corrected to normal vision. The experiments conformed to the Declaration of Helsinki and written consent was obtained from all the participants. The study was approved by the Ethics Committee of the University of Burgundy.

### Motor Task

All the participants performed a Whole Body Pointing (WBP) movement. The experimental procedures have been used and validated in previous studies [12], [23], [24], [25]. We asked participants to perform a WBP movement simultaneously with their two index fingers in order to touch two targets. The targets (4×2 cm) were separated by a distance of 0.5 m from their centres and positioned on a piece of wood. They were placed at a distance corresponding to 15% of each participant's height in the anteroposterior (AP) plane and in the vertical plane. Distances were measured from the distal end of each participant's big toe. Participants started from an upright position. Their hands were positioned so that the hypothenar eminence was in contact with the thighs. Only, the index finger remained extended while the rest of the fingers were bent. MD and target accuracy were the primary constraints imposed on the participants.

### Movement duration constraints

The WBP movement was carried out over three different durations. These were a self-selected duration (N), long duration (Lo) and shorter than normal (Sh) (without asking participants to go as fast as possible). For each movement time, all the subjects carried out about 5 preliminary unrecorded WBP movements in order to familiarize themselves with the movement and the necessary durations. This was then followed by a block of ten movements for each movement time. There was a two minutes pause between movement blocks of each duration. Each subject therefore performed a total of 30 trials.

### Data collection and processing

We used an optoelectronic device (VICON, sampling frequency 200 Hz) with three cameras in order to capture movement kinematics in 3 dimensions (3D). Twelve retro-reflective markers (0.2 m in diameter) were placed at various anatomical locations on the right side of the body (External cantus of eye, auditory meatus, acromial process, humeral condyle, ulnar styloid process, apex of the index finger, L5 vertebra, greater trochanter, knee interstitial joint space, external malleolus, fifth metatarsal head of the foot, and the middle of arm in order to have 3D with the VICON system). We used a 9 segment model similar to our previous studies of the same movement [24], [25].

All processing of the 3D marker positions was performed with custom software written in Matlab (Mathworks). Before the computation of the angular trajectories, the recorded marker position signals were low-pass filtered using a fourth-order Butterworth filter at a cut-off frequency of 10 Hz (Matlab *filtfilt* function). The filtering was followed by the use of interpolation routines (Matlab *spline* function) so that all trajectories irrespective of execution duration lay along a 200 point time base.

### Kinematic computations

**Figure 1:**
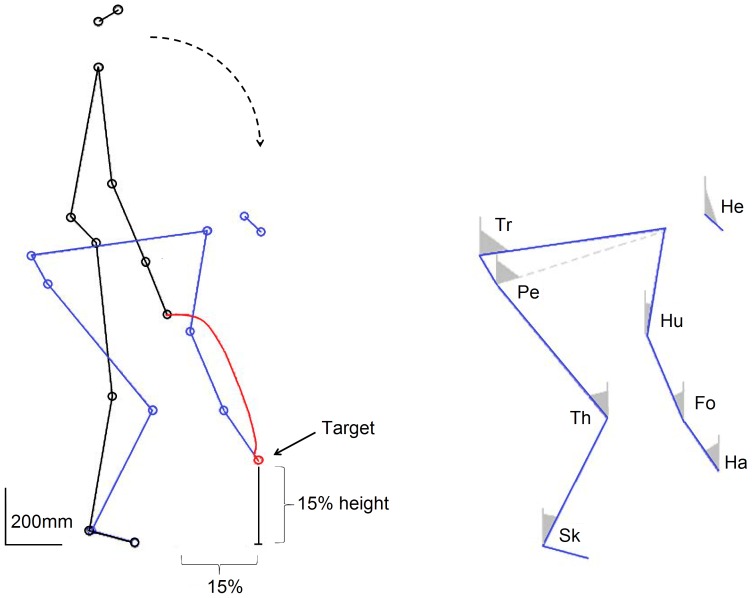
Stick diagrams. a) Stick diagrams of a whole body pointing movement to a target that is placed at 15% on the anteroposterior axis and on the vertical axis. b) The computed elevation angles for the movements were the Shank (Sk), Thigh (Th), Pelvis (Pe), Truck (Tr), Head (He), Humerus (Hu), Forearm (Fo) and Hand (Ha).

**Figure 2:**
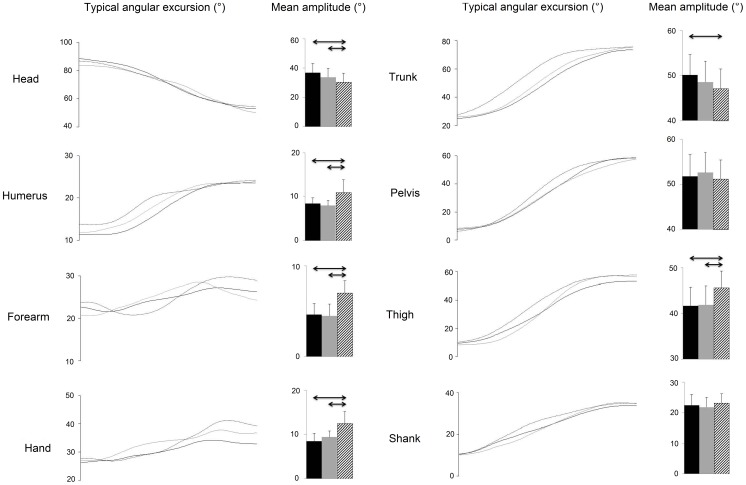
Elevation angle excursion. The kinematic trajectories of eight different elevation angles at three different movement durations for a typical subject. Beside each kinematic trace is the bar graph of the amplitudes recorded at long (Lo, black line and histogram), normal (N, grey line and histogram) and short (Sh, dotted line and hatching histogram) MDs. The amplitude of each angular displacement was defined as the absolute value of the difference between the initial and final angle. Each bar displays the mean and the SEM for all the subjects. Significantly different values are marked with an arrow.

Movement onset was defined as the time when the velocity of the finger exceeded 5% of its peak and movement cessation was noted likewise when this velocity dropped below the 5% threshold [26]. Kinematic parameters including angular displacements were computed using previously reported techniques [27], [24]. The following eight elevation angles (angle between the vertical and the segment) were calculated: Shank (Sh); Thigh (Th); Pelvis (Pe); Trunk (Tr); Humerus (Hu); Forearm (Fo); Hand (Ha) and Head (He) (figure 1b and 2). The amplitude of each angular displacement was defined as the absolute value of the difference between the initial and final angle.

### Centre of Mass analysis

We calculated the CoM displacements in 3D in order to characterize the manner in which equilibrium was managed during the WBP movement. This estimation was made from an eight-segment mathematical model using rigid segments (Head, Trunk, Thigh, Shank, Foot, Upper arm, Forearm and Hand). For this, we used the anthropometric parameters described by Winter [29] and validated by Stapley [11] and Berret [24] in previous studies of WBP movement. Stapley [11] had compared the modeled CoM and measured Centre of Pressure (CoP) position using a force platform, during quiet stance as well as the times series of measured and estimated (modeled) ground reaction forces. These studies showed that such a model provided a realistic representation of the WBP CoM position.

### Principal Component Analysis

A PCA [28] was applied to the angular displacements of the eight elevation angles. As the z scores of the elevation angles and their correlation matrices were used for this computation, the results obtained provided us with information concerning the linear correlation of these angles. In all cases, the PCA was performed separately for each individual. For the results described in section 3.2, the trials came from whole body pointing of only one duration. For the results described in section 3.6, however, the trials were either from the normal and short MDs or from the normal and long MD trials.

In all cases two principal components were found sufficient to account for more than 95% of the variance in the data. This is referred to as the variance accounted for (VAF).

### Statistical analysis

All statistical analyses were performed primarily using a repeated measures analysis of variances (ANOVA) test. The test was applied after ensuring a normal distribution of the data using the Kolgomorov-Smirnov and Lillefors test. The MDs were in all cases a repeated measures factor. The kinematic trajectory amplitudes and peak times as well as the eigen values from the PCA were also used as repeated measure factors depending on the question at hand. The post hoc tests were done using a Tukey HSD. Results were taken as statistically significant if p<0.05.

## Results

In this section we will report on the analysis of the whole body pointing (WBP) movements accomplished over different durations. We will first report on the general characteristics of the movements for the three durations - long, normal and short. The similarities between the kinematic trajectories of the movements were quantified using correlation coefficients after having reduced the dimensionality of this space with the PCA technique. Since this process revealed slight differences, we undertook a comparison of the individual kinematic trajectories at different MDs. The trajectories examined were those of the elevation angles, the joint markers and the CoM. Both the spatial and temporal characteristics of these kinematic trajectories were examined. Finally a principal components analysis using kinematic trajectories from different movement types was used to probe if common waveforms can be combined to generate the kinematic trajectories generated over different durations.

### 3.1 Movement at three speeds: General characteristics

Our first step was to verify that the subjects did indeed carry out the movements at the instructed pace. The mean MD was 1.23±0.11 s for normal MD, 1.94±0.23 s for long MD 0.77±0.06 s for short MD. The three were found to be significantly different from each other (p<0.05, repeated measures ANOVA). The mean difference between the long duration and normal movements was found to be on average slightly higher than those between the normal and short duration movements. As the subjects were asked to touch a target that was sufficiently wide and long (see Methods) all attempts to touch the target at the three different speeds were successful. The peak velocities for the normal, long duration and short duration movements were 0.88±0.08, 0.56±0.06 and 1.44±0.11 m.s^−1^ respectively.

### 3.2 Similarity of the elevation angles and their coordination for WBP at three movement durations

The trajectories of eight elevation angles were computed as described in the Methods section for whole body pointing movements executed at three different MDs. These were the head, humerus, forearm, hand, trunk, pelvis, thigh and shank elevation angles. The trajectories were normalized along a common time base as displayed in figure 2. A visual inspection of the angles revealed that their forms remained largely unaltered by the MDs.

**Figure 3:**
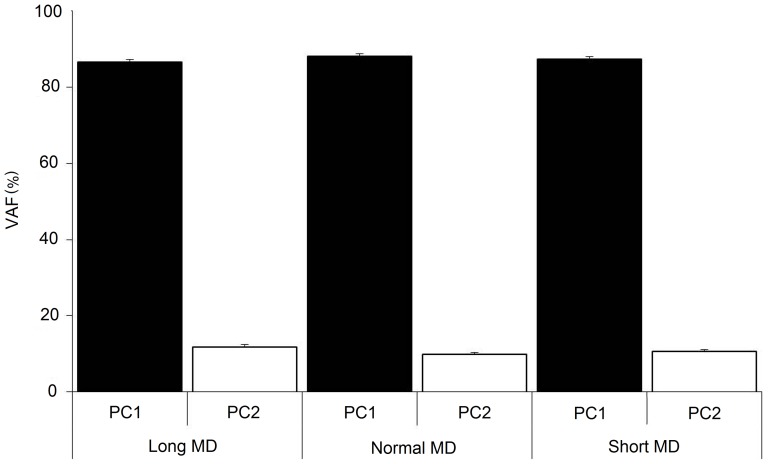
VAFs from a Principal Components Analysis of the Elevation Angles of Individual Movement Types. The eight kinematic trajectories from each type of whole body pointing could be represented using two principal components. Each bar displays the mean and the SEM for all the subjects. The VAF accounted by these components were not found to be significantly different for the different MDs (p>0.05, repeated measures ANOVA). This indicated a similar degree of correlation between the body segments at all three MDs.

**Figure 4:**
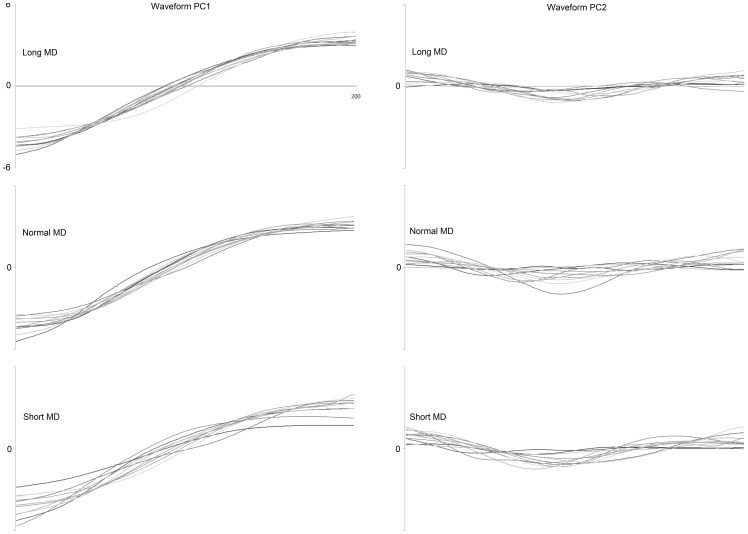
Principal component trajectories. The superimposed principal component trajectories for all the subjects at each MD. Each trace is the average for each subject. While the trajectory of the first principal component was similar for every subject and every MD, this was not the case for the second principal component (<20% VAF).

The similarity between the elevation angle waveforms was quantified by examining their correlations [30], [24], [25]. These were computed in a space of reduced dimensionality by first performing a principal components analysis on the 8 elevation angle excursions for each MD i.e. only movements of one duration were used for each PCA. For all three movement durations, two components were sufficient to capture more than 97% of the variability in the data (figure 3). The Pearson correlations between the principal components were now computed. In each case they were done by comparing the normal MD principal components with those of the short or long MDs. The mean values of these comparisons for each principal component are displayed in Table 1. They display a very high correlation between the first principal components for all three MDs. The lower correlation values when comparing the second principal component (<20% VAF) at different MDs however, indicate the presence of some small differences. The mean principal component trajectories for each individual in the study are displayed in figure 4. The trajectories displayed are for each MD. They allow a confirmation of what had been observed with Table 1 i.e. Whole body pointing over normal, short or long durations are accomplished using elevation angle excursions that are highly similar albeit with some differences.

**Table 1:** Pearson correlation.

	PC1	PC2
**Normal/Long MD**	0.97±0.02	0.51±0.12
**Normal/Short MD**	0.97±0.01	0.53±0.15

The table presents the Pearson correlation coefficient between the trajectories of the two principal components computed from each type of whole body pointing in this study. The use of the PCA allowed us to reduce the dimensionality of the space represented by the eight elevation angles. The correlations with the principal components were computed each time between the trajectories of the movements executed over normal durations with either those of the long or short duration movements. The results show that the movements were similar albeit with some small differences.

Other than permitting a comparison of kinematic trajectories in a space of reduced dimensionality, the PCA also allowed us to compare the coordination between the segments of the body for the pointing accomplished at the three different MDs. In each case the VAF by the first component exceeded at least 80%. There were no significant differences in the VAFs by the two components for the whole body pointing movement executed at the three different MDs (p>0.05, repeated measures ANOVA). This indicated that there were no significant differences in the degree of covariance between the body segments for whole body pointing at different MDs. An analysis of the loadings for each kinematic angle on the first principal component also did not reveal any significant main effects for movement duration (p>0.05, repeated measures ANOVA) (figure not included).

### 3.3 Alterations in the elevation angles

The results from the section above had established that despite an overall similarity, small differences were present in the elevation angle trajectories from whole body pointing carried out over normal, short or long durations. We proceeded to further investigate these differences by studying the individual trajectories. We first examined alterations in the amplitudes of the elevation angle excursions by comparing their values at the different MDs. For the temporal organization, we compared the time to peak velocity for each elevation angle along a normalized time axis.

#### 3.3.1 Modifications in the elevation angle amplitudes

The trajectories for all eight elevation angle trajectories were examined in order to detect differences in the amplitudes of the angular excursions (figure 2). The amplitude of each angular displacement was defined as the absolute value of the difference between the initial and final angle. First to be noted was the fact that no significant differences for this variable were found between the long and normal duration movements (p>0.05, repeated measures ANOVA, Tukey HSD). As opposed to the long-normal comparison, the amplitudes of several angles were found to be altered in the normal-short duration comparison. The angular excursions for all the focal segments were found to be increased (p<0.05, repeated measures ANOVA, Tukey HSD). In the case of the postural segment, a significant increase was observed for the thigh while a decrease in amplitude was observed for the trunk and head (p<0.05, repeated measures ANOVA, Tukey HSD). These modifications would have ensured that individuals descended lower and bent over less with their trunks for short duration movements. The descent to a lower vertical position would then have required a greater upward movement of the focal segment and hence the increased angular amplitudes of this segment.

In general, the results of this section demonstrate that the amplitudes of the elevation angles show small but significant modifications when shorter than normal MDs are employed. Such alterations in amplitude were not observed when comparing the movements carried out at normal and long durations. The results indicate that the tuning of the elevation angle amplitudes for MD is nonlinear.

#### 3.3.2 Modifications in the temporal organization of the elevation angles

**Figure 5:**
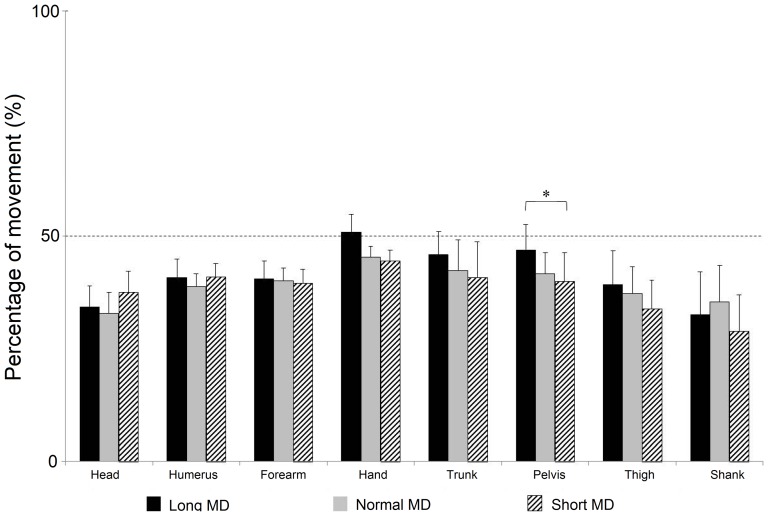
Time to peak of the elevation angle velocity profiles. The histograms display the mean and SEM of the times to peak for the velocity profiles of the elevation angle trajectories at three different MDs. This variable was normally distributed for the head, trunk, pelvis and shank elevation angles. A significant difference was only observed between the long and short MD pelvic elevation angle (p<0.05, repeated measures ANOVA, Tukey HSD posthoc).

We examined the time to peak for the velocity profiles of the elevation angle trajectories of each trial. Unlike the case for amplitude, we were able to identify only one time to peak velocity that had been modified with MD. The pelvic elevation angle for movements executed over short durations was found to acquire peak velocity significantly earlier than those executed over long durations (p<0.05, repeated measures ANOVA, Tukey HSD) (figure 5). It was feasible in this study only to examine the variables with a normal distribution. These were the times to peak velocity for the head, trunk, pelvis and shank elevation angles. With the exception of the pelvic angle, they were not found to be significantly altered by MD (p>0.05, repeated measures ANOVA, Tukey HSD posthoc).

The two previous paragraphs suggest that the temporal characteristics of the elevation angles are not tuned for MD in the same manner as their spatial characteristics. This was most notable in the case of the pelvic, trunk and head angles. While alterations with MD had been observed in the case of the amplitudes of the elevation angles of the trunk and head, there were none observed in their time to peak velocity. As opposed to significant differences in the time to peak velocity of the pelvic angle, no modifications with MD had been observed for the amplitude of this elevation angle. There was therefore a dissociation in the modifications observed as a function of MD for the temporal as opposed to spatial aspects of the elevation angle excursions.

### 3.4 Modifications in the temporal organization of the joint marker trajectories

**Figure 6:**
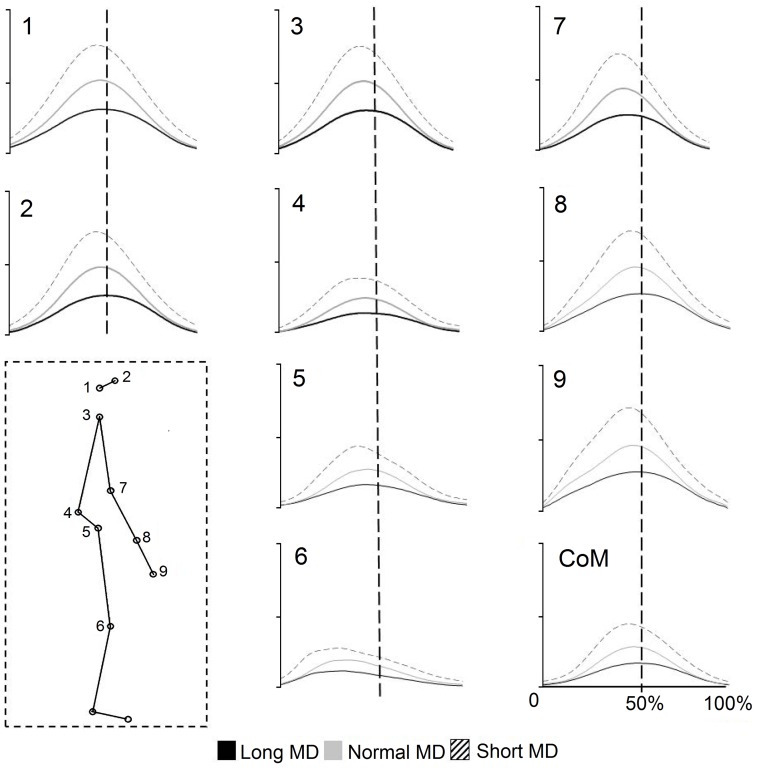
Velocity profiles of the joint markers. The inset box represents the stick diagram with the number for each marker. The velocity profiles corresponding to each marker are displayed (1–9). The last figure displays the velocity profile for the CoM. The dotted line in each case represents 50% of the movement. On the x-axis is normalized time (percentage of total movement %) and on the y-axis, the velocity (m.s^−1^).

**Figure 7:**
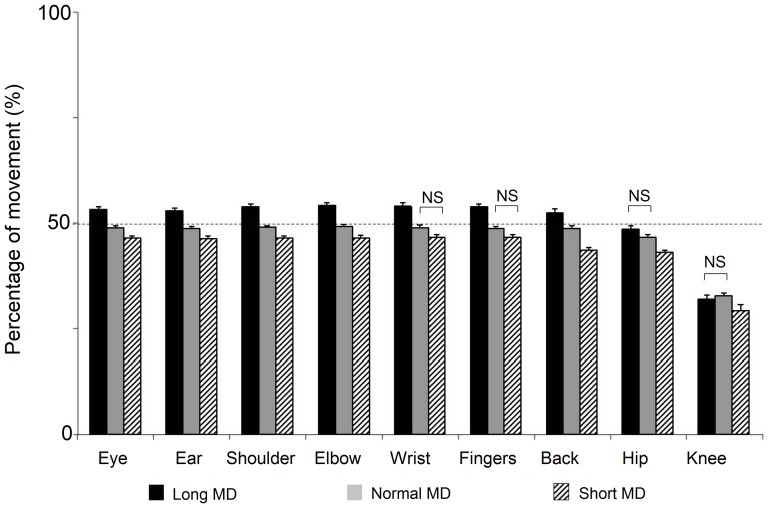
Time to peak velocity of the joint markers. After constructing the velocity profiles for the markers at each joint along a normalized time base, we examined the times at which the peak velocity occurred. The mean and SEM of these values are displayed. The dotted line marks the half-way point for each movement. With the exception of the cases marked NS, all comparisons were found to be statistically significant. The figure shows that in most cases the markers for the movements at normal durations (grey histogram) had their peak velocities close to the half-way point of the movement. The peak velocities for movements at long (black histogram) and short (hatching histogram) durations occurred slightly later or earlier respectively.

Alterations in the temporal aspects of the movement at a more integrated level were analyzed by studying the moment at which the peak velocity of each joint marker occurred on a normalized time axis. In figure 6 we display the velocity profiles for the markers at each joint. The curves displayed are the mean values from all the trials of all the individuals. A statistical comparison of the time to peak velocity was carried out. Figure 7 displays the results of this test. A significant main effect of MD was found for this variable (p<0.05, repeated measures ANOVA). The peak velocities of the markers were found on average to be phase advanced for the reaching at short durations. For normal movements they occurred on average at the mid point of the movement. Finally, the peak for long duration movements was phase delayed with respects to normal movements. With the exception of the cases marked ‘NS’, all other differences in figure 7 were found to be significant (p<0.05, repeated measures ANOVA, Tukey HSD). The most notable exception to this organization was that for the knee for which no significant differences were observed for the movements carried out over different durations (p>0.05, repeated measures ANOVA).

It is interesting to note the ordered manner in which the time to peak velocity was arranged with respects to MD for the joint marker velocity profiles. This was in notable contrast to what was observed for the velocity profiles of the elevation angles. As the trajectory of a marker is the resultant of the rotation at several joints, this provides an example of order that is emergent at a higher integrated level even when it may not be observed at the level of the individual elements composing it.

### 3.5 Alterations in the centre of mass trajectory

**Figure 8:**
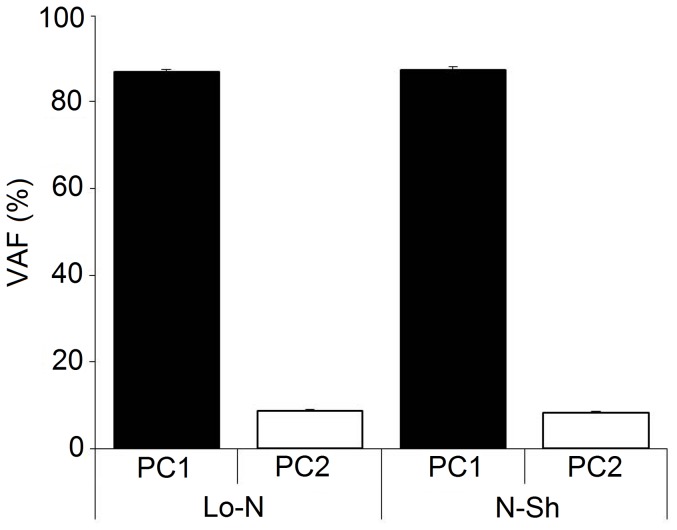
Position of the Centre of Mass (CoM). A comparison of the CoM for the movements conducted over short, normal and long MDs. All displacements were measured with respects to starting CoM positions. Significant differences for the vertical as well as anterior-posterior displacements were observed only between the movements at normal and short MDs. The inset box represents CoM trajectories for the three durations.

Other than studying the angular displacements, a more global idea of alterations in the spatial organization of the movements was obtained by examining the CoM trajectories at the three different MDs. The magnitude of the CoM displacement was studied for changes in the anterior posterior direction as well as in the vertical dimension. As in the case of the individual kinematic trajectories differences were observed mostly between the normal and short MDs (p<0.01, repeated measures ANOVA, Tukey HSD posthoc) but not between the normal and long MDs. This was true for the displacements along the anterior posterior as well as vertical directions (figure 8).

### 3.6 Combinations of kinematic synergies may be used to create movement trajectories generated over different durations

**Figure 9:**
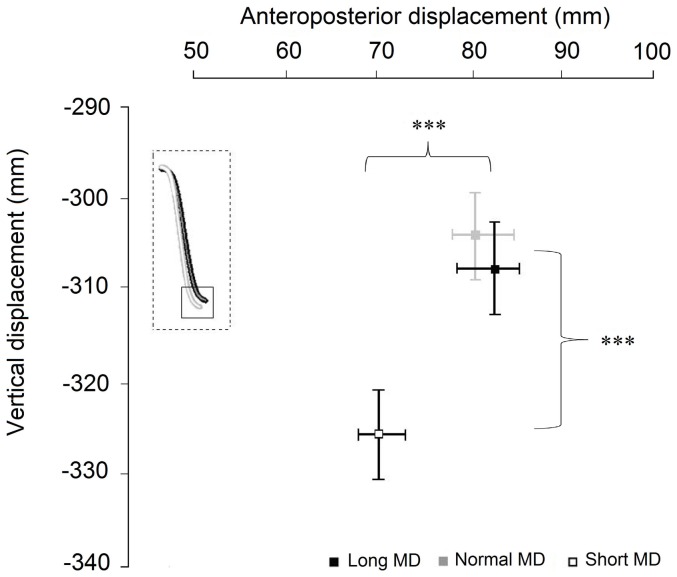
Combinations of common waveforms can be used to represent the kinematic trajectories executed over different MDs. A comparison of the VAFs of the first two principal components (PC1-PC2) when comparing the WBP at three different durations. The trajectories of the eight elevation angles were used for carrying out the PCA. In one case it was done using the long (Lo) duration and normal duration (N) trajectories together while in the second case it was done using the short (Sh) duration and normal (N) duration trajectories together. Two principal components were sufficient to represent almost all the information from movements of different durations. This suggested that combinations of common waveforms can be used to generate the trajectories for the whole body pointing over different durations. No significant differences were found between the Lo-N and Sh-N principal components (p>0.05, repeated measures ANOVA, Tukey HSD).

Results from the above sections indicated that although the kinematic trajectories utilized for carrying out the whole body pointing movements were similar, there were some differences between them. A previous study had indicated that common waveforms could be used to describe the kinematic trajectories during a reaching task at normal or short MDs [14]. Would this result be reproduced in our study? In addition, would the same be found true in the case of long MDs? In order to answer these questions, we carried out two separate PC analyses. The first one involving movements at normal and short MDs and a second one involving the kinematic trajectories from normal and long MDs. The separation of these two groups of data was carried out in order to determine if the degree of correlation was different in the two cases. A separate PCA was carried out for each individual. In both cases the first principal component alone was able to account for a big portion of the variance in the data. The mean VAF accounted for by the first component was 87.06±1.81% in the long-normal duration case and 87.63±2.11% in the short-normal duration case (figure 9). The two were not found to be significantly different (p>0.05, repeated measures ANOVA, Tukey HSD). These results with the PCA confirmed that whether over shorter or longer than normal MDs, the kinematic trajectories could be represented to a similar degree using combinations of common underlying waveforms.

## Discussion

The current study examines the manner in which a whole body pointing task is reorganized when conducted over three movement durations - viz. long, normal and short. While several previous studies have examined the alterations that took place as the movements were carried out over shorter than normal durations, there are no previous studies on the same movement conducted over durations that are longer than normal i.e. slowly. Movements carried out over long durations however are subject to several influences that may be less marked in the case of normal movements. The most obvious of these is sensorial feedback. The effort of moving slowly also alters the force that the body must use against gravity. A complete picture of ‘speed sensitive strategies’ [4], [17] can therefore only be obtained by also considering movements that are conducted over durations that are longer than normal. Finally, the influence of MD is important to take into account when examining the effects of ageing on movement.

### 4.1 General organization of movements at long, normal and short MDs

In general, our examination of the elevation angle trajectories for all three duration times after they had been normalized along a common time axis showed that they were very similar. This was borne out through several observations during the study. One of them was a visual inspection of the trajectories that we have displayed in figure 2. A second observation was the high values obtained when computing the Pearson correlation coefficients between the first principal components of the movements at each MD (Table 1).

Our result with whole body pointing therefore contributes to what has already been observed in studies of arm movements. Several theoretical arguments have been made to predict and explain why kinematic trajectories of arm movements are similar at different speeds [2], [31], [10]. Experiments have borne out many of these predictions [1], [6], [8]. While this may be true in the case of a lighter segment such as the arm, it may not have been the case for movements in which heavier segments such as the trunk are involved. In the case of reaching over to point at an object close to the ground, there could have been a re-calibration of the control strategies which optimize dynamic or static forces. A previous study comparing whole body reaching movements over normal and short MDs had shown the two to employ similar kinematic trajectories [14]. Movements over long durations however, especially with the involvement of a heavier segment such as the trunk could have potentially induced a change in strategy. We found instead that this was not the case and that similar kinematic waveforms were used for whole body pointing movements carried out over long, normal and short durations.

Explanations concerning the principles on which movement is adjusted for different movement durations are not the same. Some have offered the explanation that it is organized to minimize kinetic energy [31]. Others have explained that greater angular excursions are observed for short duration movements because of the rules underlying motor output rather than the reduction of kinetic energy [32]. Yet others have used the model of a speed invariant geometric stage between sensory input and physical execution [10]. We did not attempt in this study to find the explanation behind the adjustments that were observed. Future studies involving more theoretical and experimental work will be carried out in order to take this step.

### 4.2 Are movements at different MDs simply achieved by time scaling?

While the high degree of correlations observed between the kinematic trajectories at different MDs (Table 1) demonstrated a significant amount of similarity between them, the correlations were not perfect. A detailed comparison of the kinematic trajectories on a normalized time scale revealed that the strategy utilized was not simply one of scaling the movements in time. There were small but significant adjustments that involved both amplitude and temporal aspects of the kinematic waveforms. The temporal aspect especially is one that had not been carefully analyzed in the previous studies on the effects of speed on whole body pointing [11], [12], [13], [14]. In this section we will first discuss the modifications that had been observed in the amplitudes of the kinematic trajectories and then discuss the observed temporal adjustments.

No significant differences from normal movements were observed in the amplitudes of the elevation angles for the long duration movements. It should be noted that this was not because the difference between the normal and short durations was greater than those between the normal and long durations. It was on average 0.49 s between the means of the normal and short movement types while it was 0.67 s between the normal and long duration types. Another possible explanation for the lack of significant results concerning long duration movements might be the increased variability that is often found in slow movements. An examination of figure 2 however leads us to dismiss this as the primary explanation for the lack of significant amplitude alterations at long MDs. Especially in the case of the focal elements the mean variance was in fact higher for the short duration movements. Our results therefore strongly suggest that the tuning of angular excursion amplitudes as a function of MD in whole body movements is nonlinear. Many of these changes observed for short MDs were those that would have had the potential to increase the stability of the body's inverted pendulum configuration. When talking of angular excursions at short MDs, the increased amplitude of the thigh angular excursion concomitant with the decreased excursion for the head and trunk would have ensured a descent to a lower position and a decreased forward movement of the trunk and head axial segments. Indeed this supposition seems to be borne out with what is observed with the CoM. We found the vertical displacement of the CoM to be increased in the case of the short duration movements. There was also a significant decrease in the CoM anterior posterior displacement. These alterations would have contributed to keeping the CoM closer to the body's base of support. The increase in angular excursions with shorter MD has now been reported in several types of movements [33], [13], [34], [11]. As mentioned in the section above, there are several different explanations for these adjustments. Future theoretical and experimental studies would be required to find out which model is best able to explain our observations.

Amplitude was not the only feature that was altered as MD was increased or decreased with respects to normal pointing. We also noted the moment at which peak velocity occurred for each joint marker and for each elevation angle. In the case of the elevation angles, the time to peak velocity was only found to be significantly different for the pelvic elevation angle. For this variable, we only analyzed the elevation angles for which the time to peak velocity was normally distributed. Because of the number of variables involved in whole body pointing, posthoc pairwise comparisons in the nonparametric cases would have led to a very low value of *p* after the necessary Bonferroni corrections. Other features that distinguished the time to peak velocities of the elevation angle excursions (figure 5) from those of the joint marker trajectories (figure 7), were their non parametric distributions in some instances and finally the multidirectional nature of their modifications with MD. By this we mean that for some elevation angles, a decreased MD led to the peak velocity time occurring earlier than normal, while in other cases, the opposite was true.

The modifications observed in the spatial aspects of the elevation angles were quite different from those observed in the temporal domain. The specifics of this difference have already been provided in the results section. This therefore provides an example of dissociation between the temporal and amplitude regulation for whole body pointing. A demonstration of such a dissociation has already been made for arm pointing movements [35], [36]. Our study shows that coupling the arm movements to postural control does not alter this aspect of motor control.

As opposed to changes observed with the elevation angles, alterations in MD gave rise to very ordered shifts in the time to peak velocity of the joint marker trajectories. We observed that they had undergone phase shifts with respects to the trajectory at normal duration. The word ‘phase’ is used here as all comparisons are made using a normalised time axis. With the exception of the knee, the peak of every marker had undergone a phase advance for the short duration movements and a phase delay for the long duration ones i.e. they occurred earlier than normal for the short duration movements and later than normal for the long duration ones. The utility of such phase shifts may best be understood by noting that this means earlier transitions into the deceleration phase as the MD is decreased. This would then mean a longer period over which to break the movement when it is at a high speed. Although they was speaking of electromyographic activity, Gottlieb [4] had mentioned the use of such a ‘speed sensitive strategy’ when they reported the earlier onset of antagonist muscle activities for creating an earlier decelerating force in the case of short duration or fast movements. These observations had been made for arm movements. Our results suggest that a similar strategy is also employed when the focal element is coupled to postural displacements.

Although it was not the goal of this paper, one of the interesting results in this study was the difference in the temporal re-organization of the joint marker trajectories as opposed to those of the angular excursions. As opposed to elevation angle excursions which are created by a segment, the movements of joint markers are the result of the angular excursions of several segments, not all of which are displaced in the same direction. This sort of difference between elevation angle excursions and joint marker trajectories is likely to be more marked in the case of whole body movements than for isolated arm movements. These individual changes at the level of the joints then gave rise to the sort of changes described in the paragraph above i.e. those that lead to earlier deceleration for shorter MD movements.

### 4.3 High covariation between the body segments for all MDs

The degree of covariation between the segments was quantified using a PCA. In all cases two principal components were sufficient to capture practically all the variance in the data, hence indicating a high degree of correlation between the angular displacements of all the body segments. Despite significant differences in the kinematic trajectories with MD, the coordination between the segments remained similarly high for all the movements. This suggested that modifications in any body segment were coordinated with similar modifications in the remaining segments.

The VAF by the first principal component in our study is slightly lower than what had been observed in the study of Thomas et al [14] when analyzing fast reaching movements or by Alexandrov et al [34] when looking at trunk bending movements of various durations. The most likely reason for this might have been the lower number of variables involved in the two cited studies.

### 4.4 Combinations of a few kinematic synergies can generate movement trajectories at different MDs

A principal components analysis using kinematic trajectories of different MDs showed that common waveforms could be used to represent all the movements. We did not perform an analysis with all three movement types at once. It was done instead with the long-normal and short-normal kinematic trajectories separately. This was done in order to probe if the inter-speed segmental covariation might be different for these two groups. Two principal components were sufficient to account for more than 95% of the variance in the data in both cases. No significant differences were found between the VAF by the first principal component in either group. This was also the case for the second principal component component. The ability to represent with two components, the trajectories from different movement types, indicates that they could be generated using combinations of a few common waveforms.

### 4.5 Unintended modifications in movement durations

It should be noted that movement duration is not always something that is intentionally controlled. It has been found to vary for the same target location despite the lack of any explicit instructions or incentives to do so [36]. It can also change unintentionally when the characteristics of the movement trajectory change [37]. Fitt's law [38] concerning the change of movement speed with alteration in stimulus position or dimensions is one of the most studied examples in motor control [33]. We were unable in this study to make any claims concerning unintentional alterations in movement duration as all subjects had been given explicit instructions concerning this variable. It will be interesting in the future to examine how movement duration may be implicitly altered as different aspects of the whole body pointing such as distance or directions are modified.
